# Tuberculous meningitis

**DOI:** 10.1002/ccr3.6865

**Published:** 2023-01-16

**Authors:** Shinnosuke Fukushima, Kazuki Ocho, Koji Fujita, Hideharu Hagiya, Fumio Otsuka

**Affiliations:** ^1^ Department of General Medicine Okayama University Graduate School of Medicine, Dentistry and Pharmaceutical Sciences Okayama Japan; ^2^ Department of General Medicine and Infectious Diseases Tsuyama Chuo Hospital Tsuyama Japan; ^3^ Department of Internal Medicine Ishikawa Hospital Tsuyama Japan

**Keywords:** basal meningitis, cerebral infarction, miliary tuberculosis, tuberculous meningitis

## Abstract

Tuberculous meningitis is possibly complicated with multiple cerebral infarctions and basal meningitis, and the mortality and neurological prognosis is reportedly poor. This case suggested that clinicians should consider tuberculous meningitis as a differential diagnosis of patients with disturbed consciousness in an aging country Japan.

## 1 CASE

**FIGURE 1:**
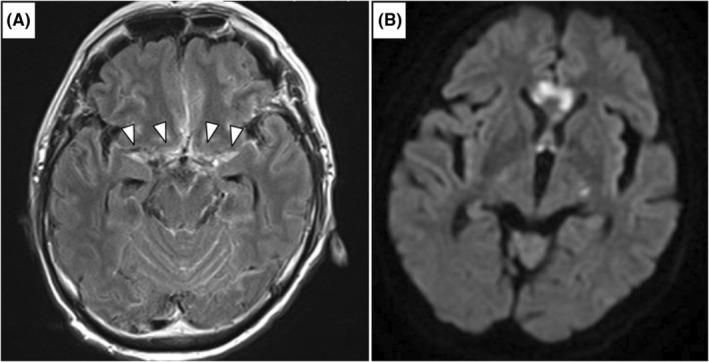
Head magnetic resonance imaging. The basal meningeal enhancement was observed (A; gadolinium‐enhanced fluid‐attenuated inversion recovery image: arrow heads), along with multiple infarctions mainly at the corpus callosum (B; diffusion‐weighted image). Tuberculoma, hydrocephalus, and vasculitis were not found.

A 72‐year‐old previously healthy man with a medical history of hypertension was hospitalized with fever and confusion. Chest computed tomography (CT) images revealed diffuse granules in the lungs, suggesting miliary tuberculosis. Cerebrospinal fluid (CSF) analysis showed elevated levels of cell count (58 /μl), protein (374 mg/dl), and adenosine deaminase (17 U/L), with low glucose levels (32 mg/dl; serum glucose level at 169 mg/dl). Culture and PCR testing for *Mycobacterium tuberculosis* in the CSF provided negative results, while sputum testing showed positivity. Contrast‐enhanced magnetic resonance imaging (MRI) revealed meningeal enhancement from the basilar portion of the cerebrum to the sylvian fissure and multiple infarctions **(**Figure 1 **)**. The patient was diagnosed with miliary tuberculosis and tuberculous meningitis (TBM), was administered antituberculosis agents along with systemic corticosteroid therapy, and finally survived with dysphagia and dysarthria as sequelae.

TBM is complicated with multiple infarctions and basilar meningitis, resulting in high mortality and neurologic sequelae. The typical findings of TBM in MRI are meningeal enhancement, particularly at the basilar meninges and the sylvian fissure,^1^ and vasculitis‐related infarction at the circle of Willis.^2^ TBM should be listed as a differential diagnosis for patients with disturbed consciousness, for which MRI should be performed.

## AUTHOR CONTRIBUTIONS

**Shinnosuke Fukushima:** Writing – original draft. **Kazuki Ocho:** Writing – review and editing. **Koji Fujita:** Writing – review and editing. **Hideharu Hagiya:** Writing – review and editing. **Fumio Otsuka:** Writing – review and editing.

## ACKNOWLEDGEMENTS

We would like to thank Editage (www.editage.jp) for English language editing.

## CONFLICT OF INTEREST

No authors have any competing interests in this case.

## INFORMED CONSENT

Written informed consent was obtained from the patient to publish this case report in accordance with the journal's patient consent policy.

## Data Availability

None.
